# Tailoring Bandgap of Perovskite BaTiO_3_ by Transition Metals Co-Doping for Visible-Light Photoelectrical Applications: A First-Principles Study

**DOI:** 10.3390/nano8070455

**Published:** 2018-06-21

**Authors:** Fan Yang, Liang Yang, Changzhi Ai, Pengcheng Xie, Shiwei Lin, Cai-Zhuang Wang, Xihong Lu

**Affiliations:** 1 State Key Laboratory of Marine Resource Utilization in South China Sea, Hainan University, Haikou 570228, China; 18789276621@163.com (F.Y.); changzhiai@outlook.com (C.A.); jxxpc199406@163.com (P.X.); 2 College of Materials and Chemical Engineering, Hainan University, Haikou 570228, China; yl5923@sina.com.cn; 3 Ames Laboratory, U. S. Department of Energy, and Department of Physics and Astronomy, Iowa State University, Ames, IA 50011, USA; wangcz@ameslab.gov; 4 School of Chemistry, Sun Yat-Sen University, Guangzhou 510275, China; Luxh6@mail.sysu.edu.cn

**Keywords:** BaTiO_3_, co-doping, first-principles, photoelectrical

## Abstract

The physical and chemical properties of V-M″ and Nb-M″ (M″ is 3d or 4d transition metal) co-doped BaTiO_3_ were studied by first-principles calculation based on density functional theory. Our calculation results show that V-M″ co-doping is more favorable than Nb-M″ co-doping in terms of narrowing the bandgap and increasing the visible-light absorption. In pure BaTiO_3_, the bandgap depends on the energy levels of the Ti 3d and O 2p states. The appropriate co-doping can effectively manipulate the bandgap by introducing new energy levels interacting with those of the pure BaTiO_3_. The optimal co-doping effect comes from the V-Cr co-doping system, which not only has smaller impurity formation energy, but also significantly reduces the bandgap. Detailed analysis of the density of states, band structure, and charge-density distribution in the doping systems demonstrates the synergistic effect induced by the V and Cr co-doping. The results can provide not only useful insights into the understanding of the bandgap engineering by element doping, but also beneficial guidance to the experimental study of BaTiO_3_ for visible-light photoelectrical applications.

## 1. Introduction

Perovskites have been extensively studied because of their unique structure and properties. Over the past decade, organic-inorganic hybrid perovskite iodides have received considerable attention due to their high efficiency in the photovoltaic process [1]. However, stability is a major issue, hindering a large-scale commercialization of the perovskite for photovoltaic applications [2,3,4,5]. To overcome this problem, inorganic perovskites, especially ferroelectric oxide perovskites, have attracted much current interest [6]. For example, Bi_2_FeCrO_6_ as a double perovskite material with its bandgap tunable by bandgap engineering has been reported [7]. [KNbO_3_]_1−x_[BaNi_1/2_O_3−δ_]_x_ has a bandgap adjustable in the range of 1.1–3.8 eV [8]. Other perovskites, such as BaTiO_3_ [9,10,11], BiFeO_3_ [12,13,14,15], LiNbO_3_ [16,17,18,19] and PbTiO_3_ [20,21,22] have also been extensively studied.

Inorganic oxide perovskites ABO_3_ have been investigated for photovoltaic applications due to their tunable bandgap [23], wide range of possibilities for switchable component [24], low cost [8], and high chemical stability. Recently, the potential of this class of materials for photovoltaic applications has been demonstrated by several theoretical and experimental studies. However, it is also known that these materials usually have limited light absorption due to the wide bandgaps and poor electrical conductivity [25]. Currently, the most common method used to overcome these problems is doping with either metal or non-metal elements. In terms of the feasibility and effectiveness, element doping is one of the most important strategies to regulate the bandgap of oxide perovskites at the atomic scale structure modification.

In one of our previous papers, we have systematically investigated the effects of single element doping with various metals on the performance of BaTiO_3_ [26]. We showed that impurity levels could be introduced in the bandgap by the metal doping. These impurity levels can act as recombination centers for electrons and holes, which are harmful for the performance of the materials for photovoltaic applications. In general, wide bandgap semiconductors with doping may exhibit some unwanted properties or behaviors [27]: (i) desirable impurities may have limited solubility; (ii) for those impurities that have sufficient solubility, they may form deep levels that are not effective for carrier transitions; and (iii) spontaneous formation of compensating defects. In the literature, it has been shown that some of these unwanted features could be avoided by co-doping two different types of elements [28]. Ishii et al. [29] reported that Cr^3+^ and Ta^5+^ co-doped SrTiO_3_ can improve photocatalytic activities. Kako et al. [30] reported a visible-light sensitive TiO_2_ with Fe-Ta co-doping ((Fe,Ta)_x_Ti_1−x_O_2_, 0 ≤ x ≤ 1) that has a higher photocatalytic activity than the Fe^3+^ doped TiO_2_. Masahiro et al. [31] also reported that N and La co-doped SrTiO_3_ had better visible-light absorption (≥400 nm) compared with the pure SrTiO_3_.

In this paper, we screened out the suitable co-doped elements by studying the defect formation energy and electronic properties of the transition metals co-doping on the Ti-site in BaTiO_3_. In comparison to the results of single doping, we evaluated the synergistic effects between the two co-doping elements and tried to use co-doping to overcome the shortcomings of single metal doping and avoid the formation of the deep levels in the bandgap, so as to promote the separation of photogenerated electron-hole pairs and expand the visible-light absorption range. Such information can provide beneficial guidance for the experimental regulation of the BaTiO_3_ bandgap at atomic scale for visible-light utilization.

## 2. Calculation Details

**Figure 1:**
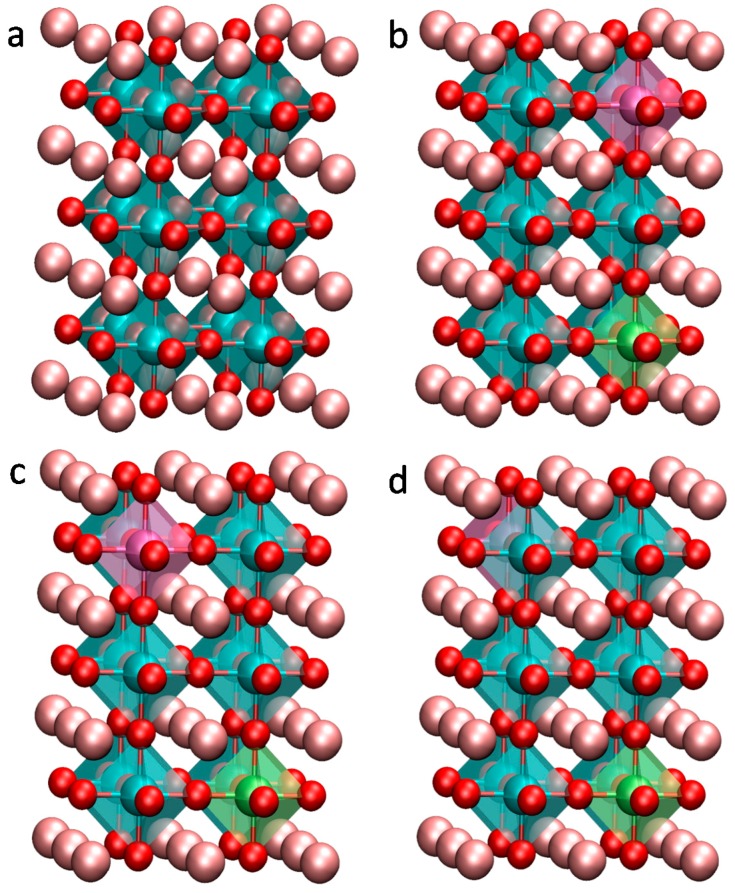
Models for the calculation. (**a**) the structure of 2 × 2 × 3 supercell model of BaTiO_3_; (**b**–**d**) configurations for transition metals co-doping in BaTiO_3_. The purple and green spheres indicate the transition metal dopants. The pink, blue and red spheres indicate Ba, Ti and O atoms, respectively.

In order to avoid the strong interaction between the co-doping elements and obtain a relatively reasonable doping concentration, the co-doping system was modelled using a 2 × 2 × 3 (60 atoms) supercell of the cubic BaTiO_3_ unit cell, as shown in Figure 1a. The dopant concentration was 3.3%. Two Ti atoms are substituted by two different transition metals (M′ and M″) and the chemical formula can be expressed as Ba_8_ (M′M″) Ti_6_O_24_, where M′ refers to V or Nb, while M″ includes 3d transition metals (Cr, Mn, Fe, Co and Ni) and 4d transition metals (Mo, Tc, Ru and Pd). There are three kinds of possible substitution. These doping situations are schematically shown in Figure 1b–d and the lowest energy situation is described in Figure 1b. The pure BaTiO_3_ system was modelled using a 1 × 1 × 1 supercell. To keep the doping concentration similar between the single doping and co-doping systems, the single doping system was modelled using 2 × 2 × 2 supercell. The atomic percentage of the impurity was 2.5%.

The spin-polarized first-principles density functional theory (DFT) calculations were performed using the Vienna ab initio simulation package (VASP, Version 5.2, Materials Design Inc., Vienna, Austria) [32,33,34]. We used the generalized gradient approximation (GGA) [35] with the Perdew–Burke–Ernzerhof formulation (PBE) [36] to treat the exchange and correlation energy. The plane-wave energy cutoff was set as 500 eV within the projector-augmented-wave method (PAW). The Monkhorst–Pack scheme K-points mesh was set as 4 × 4 × 2 [37]. The structure optimization was performed until the residual force was less than 0.01 eV/Å on every atom [38]. In the process of calculating the band structure, the special points are X→R→M→Г→R (1 × 1 × 1 and 2 × 2 × 2 supercell) and Г→F→Q→Z→Г (2 × 2 × 3 supercell). The special points coordinates are shown in Table S1.

## 3. Results and Discussion

### 3.1. Structure

The calculated lattice parameter of pure BaTiO_3_ is 4.03 Å, which is in good agreement with the experimental value of 4.0 Å (within an error of 0.07%) [39]. The lattice parameter of the transition metal co-doped BaTiO_3_ was also obtained after geometric structure optimization. The volume of the co-doped structure is not only dependent on the radii of the doped elements, but related to the interaction between atoms. As shown in Figure S1, after co-doping, the lattice constants a and b decrease slightly (except V-Ni and Nb-Ni) while c increases and oscillates. It indicates that the crystal cell is slightly enlarged in the c direction. As shown in Tables S2 and S3, the interatomic bond length varies due to the lattice distortion after co-doping. This means that the center of the positive charge after metal co-doping into the octahedral does not coincide with the center of the negative charge, resulting in the internal dipole moment. Since different elements co-doping causes a different interaction force, the deformation degree of the octahedral is different and the lattice constant accordingly changes. In this study, we chose V or Nb as one of the co-doping elements. Namely, the co-doping systems can be divided into two groups: V-M″ and Nb-M″. On one hand, V and Nb are typical 3d and 4d transition metals, respectively. On the other hand, the locations of V and Nb in the periodic table of elements are close to Ti, and their atomic radii are similar. Thus, it is more suitable for V and Nb to replace Ti. In addition, the radius of V is less than Nb, so the volume of the V-M″ co-doping system is smaller than that of the Nb-M″ co-doping system. Also, the volume of the 3d transition metals co-doping systems is usually smaller than that of the 4d transition metals co-doping systems.

### 3.2. Defect Formation Energy

**Figure 2:**
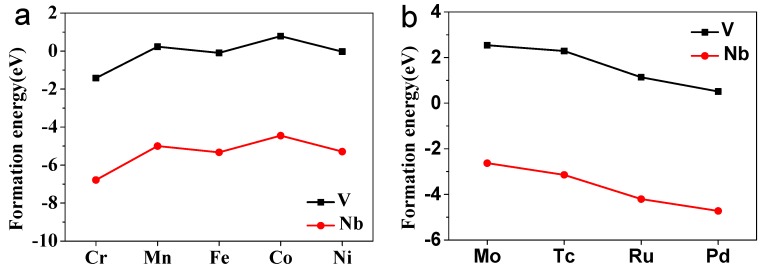
The defect formation energies of transition metals co-doped cubic BaTiO_3_. (**a**) V or Nb with 3d metal M″ (Cr, Mn, Fe, Co, and Ni); (**b**) V or Nb with 4d metal M″ (Mo, Tc, Ru, and Pd).

The defect formation energy ($E_{F\mathrm{orm}}$) of transition metals co-doping BaTiO_3_ can be calculated according to the formulas:

(1) $$E_{F\mathrm{orm}}=E_{co-doped}-E_{pure}-\mu_{M\prime }-\mu_{M\prime \prime }+2\mu_{Ti}$$

(2) $$\mu_{M\prime }{}_{/M\prime \prime }=\left(\mu_{{\left({{}_{M\prime }}_{/M\prime \prime }\right)}_{m}O_{n}}-n\mu_{O}\right)/m$$

(3) $$E_{TiO_{2}}=\mu_{Ti}+2\mu_{O}$$

where *E_co-doped_* is the energy of the co-doped BaTiO_3_ system, *E_pure_* is the total energy of pure BaTiO_3_ supercell, *μ_M_*_′_ and *μ_M_*_″_ are the chemical potentials of the co-doping metal elements, and *μ_Ti_* is the chemical potential of Ti. The *μ_M_*_′_ and *μ_M_*_″_ are defined and calculated from Equation (2), and the *μ_Ti_* is defined by Equation (3). The $\mu_{{\left({{}_{M\prime }}_{/M\prime \prime }\right)}_{m}O_{n}}$ is the energy of the most stable oxide of the doping metal atom. $\mu_{O}$ can be calculated from the ground state energy of O_2_. Under the equilibrium condition, the concentrations of point defects are controlled by their formation energies, which rely on the chemical potentials of impurity atoms and host [6]. Thus, the smaller the defect formation energy value is, the more stable the co-doped BaTiO_3_ is. The defect formation energies of all co-doped BaTiO_3_ are shown in Figure 2. In comparison, the defect formation energies of Nb-M″ co-doping are energetically more favorable than V-M″ co-doping. It is difficult to find the tendency of *E_Form_* with the increase of the atomic number of M″. The formation energies of V-M″ (Cr, Fe and Ni) and Nb-M″ (Cr, Mn, Fe, Co, Ni, Mo, Tc, Ru, and Pd) are negative, which means that the co-doping system is energetically favorable and could be easily prepared in the experiment. From Tables S2 and S3, the average bond length of Nb-O appears identical with that of Ti-O. Therefore, the influence of Nb doping into the structure of BaTiO_3_ is smaller, and thus the formation energy is smaller and the structure is more stable. The deformation of the octahedral in the V-M″ co-doping system is thus more serious, corresponding to the stronger internal stress and unstable structure.

### 3.3. Electronic Properties

**Figure 3:**
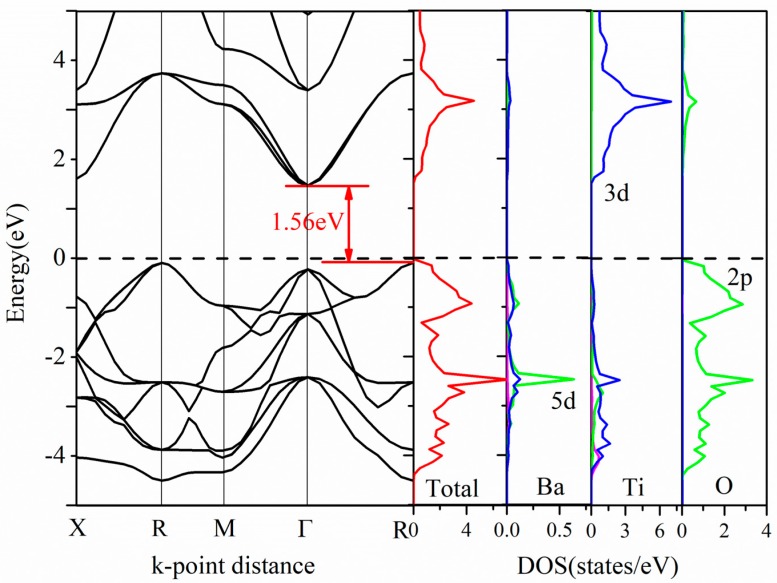
Band structure, total and partial density of states of pure cubic BaTiO_3_.

Figure 3 shows the calculated band structure and density of states (DOS) of pure cubic BaTiO_3_. The band structure shows an indirect bandgap of 1.56 eV, which is smaller than the experimental value (3.2 eV) as the small calculated bandgap is a systematic error in the DFT calculation [40]. However, the DFT calculation can still give a reliable description of the result on the trend of bandgap variation due to doping. The valence band maximum (VBM) is mainly derived from the Ti 3d and O 2p states [41]. The conduction band minimum (CBM) is derived from the Ti 3d states. Ba does not contribute to the VBM and CBM, although it does provide electrons to balance the system charge [42]. Therefore, the bandgap value of BaTiO_3_ depends on the relative energy positions of the Ti 3d and O 2p states. However, as a light absorbing material, the bandgap of BaTiO_3_ is too large to efficiently absorb the visible light.

In order to clarify the co-doping effects with different transition metals on the electronic properties of the cubic BaTiO_3_, we systematically and carefully compared the band structures and density of states of the different co-doping systems. The co-doping systems can be divided into two types: BVM″TO and BNbM″TO. M″ includes 3d transition metals (Cr, Mn, Fe, Co, and Ni) and 4d transition metals (Mo, Tc, Ru, and Pd). Accordingly, the classified discussion is conducted in detail as follows. Although there exist the spin channels, the magnetic properties of the system have not been discussed in the article. The main reason is that this article mainly focuses on the change of the bandgap in the co-doped system and the effect on the absorption of light.

#### 3.3.1. V and 3d M″ Co-Doping (Cr, Mn, Fe, Co and Ni)

**Figure 4:**
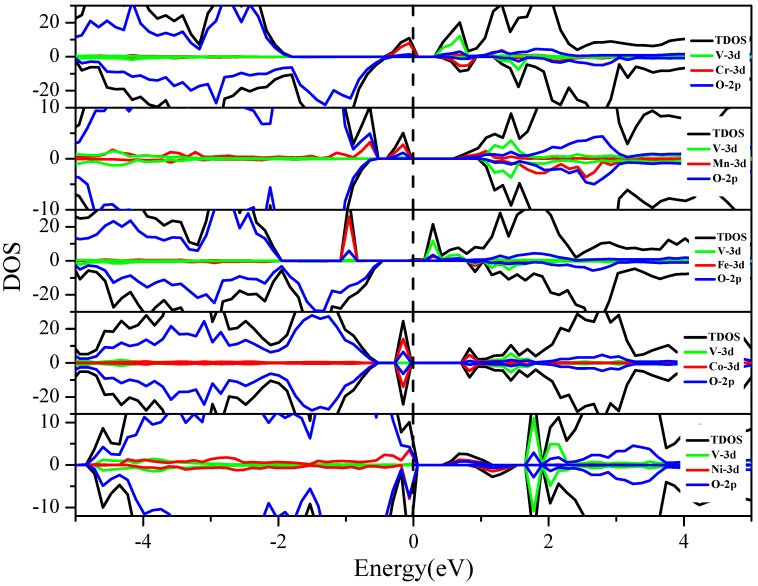
The total and partial density of states of the V-M″ (3d metal elements: Cr, Mn, Fe, Co, and Ni) co-doped BaTiO_3_. The black dashed line indicates the position of the Fermi level.

Figure 4 reveals that the bandgap of BaTiO_3_ after co-doping is narrowed due to the introduction of the impurity energy levels (IELs). It is noticed that BaTiO_3_, after V-M″ co-doping, exhibits ferromagnetism, except for V-Co co-doping. The ferromagnetism caused by transition metal doping is also found in other doped semiconductors, such as V-Cr co-doped ZnO [43], Mn doped GaN [44], and Mn doped AIN [45]. The majority spin (up-spin) DOS has a smaller bandgap than the minority spin (down-spin) DOS. The bandgaps on both spin states are formed by the interaction of the V 3d or M″ 3d states with the O 2p states. The electronic structure of BaTiO_3_ is significantly modified by the dopants. The V 3d states are located at the bottom of the conduction band (CB), while the 3d states of Cr, Mn, Fe, Co, and Ni show up at the top of valence band (VB) (V-Cr, V-Mn and V-Fe co-doping) or in the middle of the bandgap (V-Co and V-Ni co-doping). Thus, the energy states from the dopants form new CBM, VBM, or a transition state in the bandgap, leading to a relatively narrow bandgap. Figure 4 shows that the IELs (from Cr, Mn, Co, and Ni) move from left to right, which means the highest occupied energy level of the 3d states is arranged in the order of Cr < Mn < Co < Ni from lower to higher energy, consistent with the number of the d electrons in these atoms.

**Figure 5:**
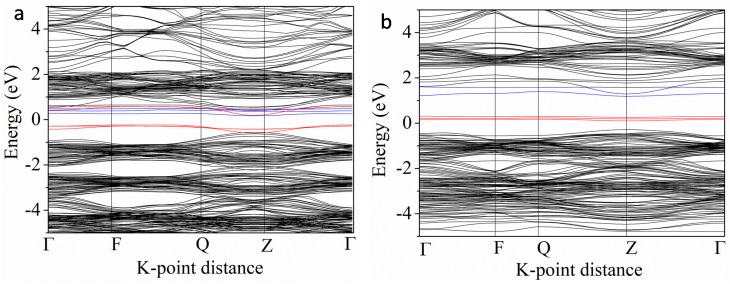
The band structures of the V-Cr (**a**) and V-Co (**b**) co-doped BaTiO_3_. The red lines indicate Cr 3d states or Co 3d states. The blue lines indicate V 3d states.

For further analysis of the effect of IELs in the co-doping systems, we compared the band structures of the V-Cr (a) and V-Co (b) co-doping systems (Figure 5). The bandgaps of V-Cr (a) and V-Co (b) are 0.4 eV and 1.46 eV, respectively. The V 3d and Cr 3d states make the VBM and CBM shift to the middle and result in a smaller bandgap. But the Co 3d states are located in the middle of the bandgap, forming an intermediate level. Such a level in the bandgap usually plays two roles. One is to form a step for the electron transition and the other is to work as the electron-hole recombination center when the doping concentration is too high. The former is beneficial to visible light absorption, but the latter hinders electron-hole separation. Therefore, combining Figure 4 and Figure 5, the results show that V-Cr, V-Mn, and V-Fe co-doping should have suitable bandgap and better visible light absorption than V-Co and V-Ni.

#### 3.3.2. V and 4d M″ Co-Doping (Mo, Tc, Ru and Pd)

**Figure 6:**
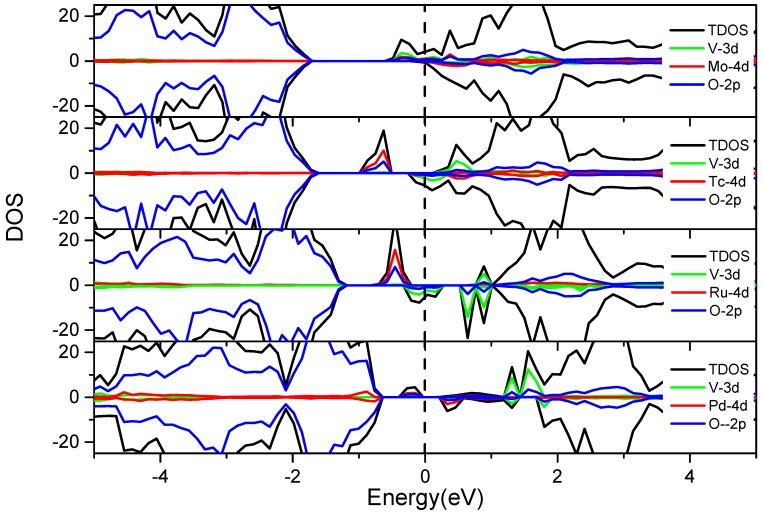
The total and partial density of states of the V-M″ (4d metal elements: Mo, Tc, Ru and Pd) co-doped BaTiO_3_. The black dashed line indicates the position of the Fermi level.

As shown in Figure 6, the IELs introduced by V-4d M″ co-doping emerges in the middle of bandgap and CB. Compared with Figure 4, the IELs of the 4d metals are even closer to the CB than the IELs of 3d metals. The reason is that the 4d states have higher energy than the 3d states of the transition metals. Therefore, the IELs of V co-doping with the 4d transition metals are closer to the conduction band and belong to the significant n-doping. The Fermi level (E_F_) gradually shifts from the conduction band to the valence band with the increase of the atomic number of the M″ elements. The definition of the Fermi level is the highest energy level of the electron at the absolute zero degree, which corresponds to the energy state at 0 eV. It can act as a reference to discuss the effects of the transition metal co-doping on the band edge modification and the bandgap variation. The IELs in the bandgap and near the bottom of the CB partially (V-Tc, V-Ru and V-Pd co-doping systems) or wholly overlap with the CBM (V-Mo co-doping system). Both cases can lead to the CBM downward shifting, while the valence bands remain barely unchanged upon the co-doping. The Fermi levels of the V-M″ (Mo, Tc, Ru and Pd) co-doping systems pass through the IELs, that is to say, the electrons can occupy the IELs below the Fermi level in the ground state. Due to the small distance between this energy level and the bottom of the CB, electrons can be excited by absorbing small photon energy [46,47].

#### 3.3.3. Nb and 3d M″ Co-Doping (Cr, Mn, Fe, Co and Ni)

**Figure 7:**
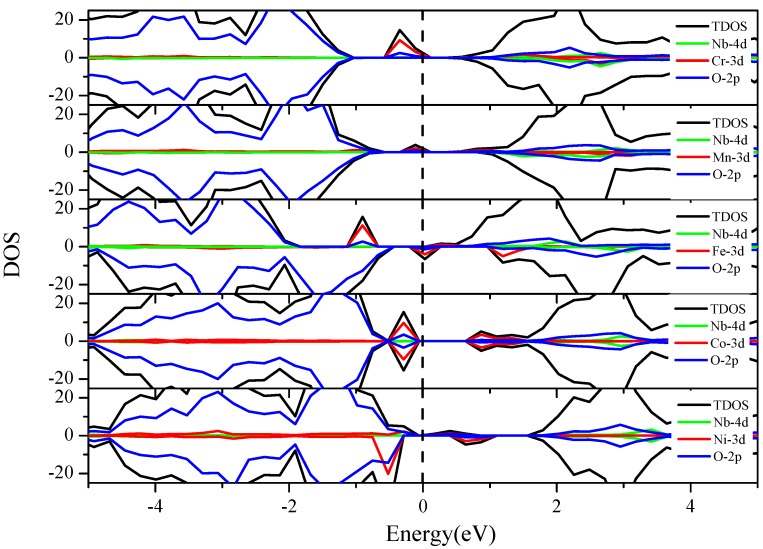
The total and partial density of states of the Nb-M″ (3d metal elements: Cr, Mn, Fe, Co, and Ni) co-doped BaTiO_3_. The black dashed line indicates the position of the Fermi level.

Figure 7 shows that the effects of this series of co-doping are similar to the V-M″ (Cr, Mn, Fe, Co and Ni) co-doping described in Figure 4. The only difference is that the energy of the Nb 4d states is higher than that of the V 3d states. Consequently, the V 3d states are located at the CBM but the Nb 4d states are in the upper CB. Therefore, the Nb 4d states have little effect on the bandgap and there is no synergistic effect between the co-dopants. The Fermi level moves from CB to VB with the increase of the atomic number of M″ elements. The Cr, Mn, and Ni 3d states occur in the middle of the bandgap and become the intermediate level. The intermediate state might be beneficial to the electron transition under visible-light irradiation. However, it might not be conducive to the effective separation of the electron-hole since it could also act as the recombination centers on the other hand. In addition, the Fe and Co 3d states are divided into two parts, where the high energy part is weak at the bottom of the CB, and the low energy part is strong, which is located at the top of the VB. Both cases can lead to the bandgap narrowing and are beneficial to visible-light absorption.

**Figure 8:**
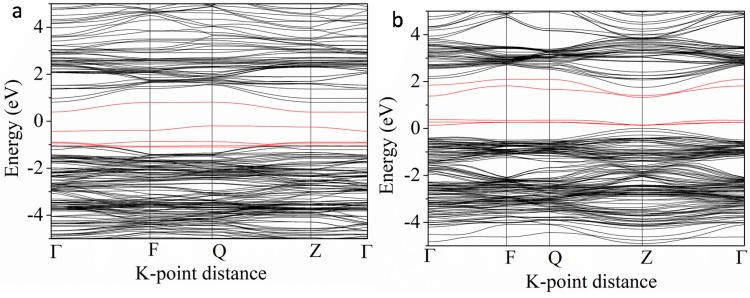
The band structures of Nb-Mn (**a**) and Nb-Co (**b**) co-doped BaTiO_3_. The red lines indicate Mn 3d states (**a**) and Co 3d states (**b**).

In these Nb-M″ co-doping systems, we chose two typical doping forms, the Nb-Mn and Nb-Co co-doping systems, to further investigate their band structures, as shown in Figure 8. The bandgaps are found to be 1.2 and 0.96 eV, respectively. In the Nb-Mn co-doping system, the IELs in the middle of bandgap are contributed by the Mn 3d states. However, in the Nb and Co co-doping system, the Co 3d and O 2p states form the p-d hybridization and locate above the original VB or partly coincided with the bottom of the CB. Overall, both co-doping could reduce the bandgap effectively but with different causes. It is also necessary to consider that Nb and Mn heavy co-doping might lead to a serious recombination due to the IELs in the bandgap.

#### 3.3.4. Nb-4d M″ Co-Doping (Mo, Tc, Ru and Pd)

**Figure 9:**
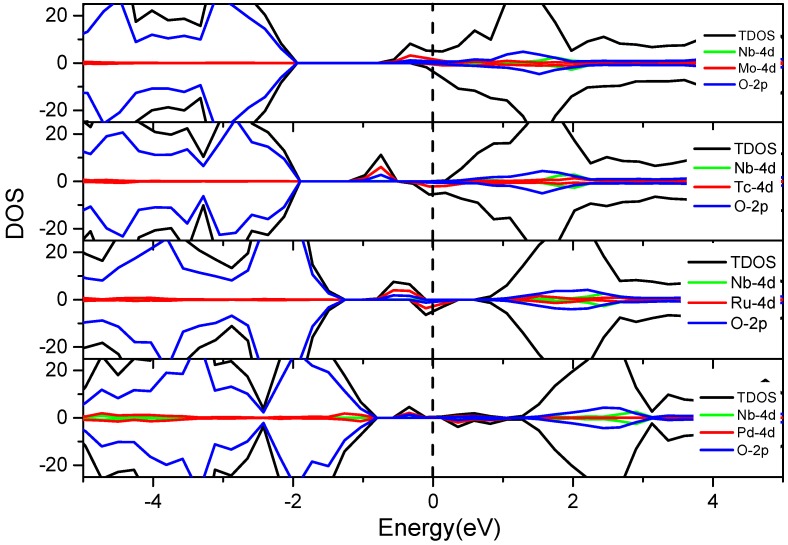
The total and partial density of states of the Nb-M″ (4d metal elements: Mo, Tc, Ru and Pd) co-doped BaTiO_3_. The black dashed line indicates the position of the Fermi level.

As shown in Figure 9, Nb-Mo, Nb-Tc, Nb-Ru, and Nb-Pd co-doping have very little influence on the top of valence band, but it changes the distribution of the electron states at the bottom of the conduction band. The Nb 4d states are in the middle of the conduction band, while the Mo, Tc, Ru, and Pd 3d states are located at the conduction band bottom or in the bandgap. Hence this series of co-dopings have no synergistic influence, and the doping effects around the bandgap are similar to the individual Mo, Tc, Ru, or Pd doping.

Generally, according to the results above, transition metal co-doping can have three effects on reducing the bandgap. Firstly, the IELs appear below the CB, partially or wholly overlap with the bottom of the CB, and eventually cause the CBM to move downward. Secondly, the IELs are located above the VB, overlap with the top of VB in different degrees, and make the VBM move up. Thirdly, the IELs lie in the middle of the bandgap to form the intermediate energy level. In the actual co-doping system, two or more effects can coexist to modify the energy bandgap.

**Figure 10:**
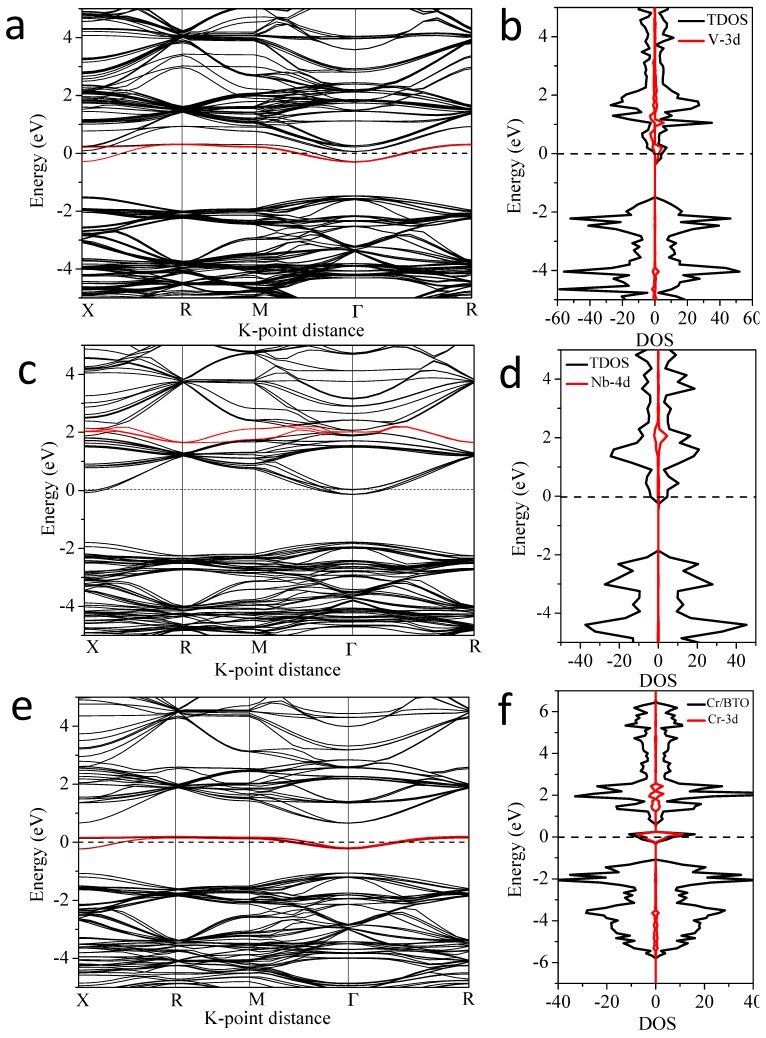
Band structures and total and partial density of states (DOS) of (**a**,**b**) Ba_8_Ti_7_VO_24_, (**c**,**d**) Ba_8_Ti_7_NbO_24_, and (**e**,**f**) Ba_8_Ti_7_CrO_24_.

In order to further study the difference between the single doping and co-doping systems, we calculated the band structures and total and partial DOS of V doped BaTiO_3_, Nb doped BaTiO_3_, and Cr doped BaTiO_3_, as shown in Figure 10. The band structures clearly show that V doping reduces the bandgap to 1.15 eV since the V 3d states appear at the bottom of CB and form a new CBM. But the bandgap after Nb doping is 1.63 eV, which is even a bit larger than pure BaTiO_3_ (1.56 eV). The reason is that the Nb 4d states are located in the CB and far away from CBM, which have little effect on the bandgap. Thus, in the Nb-M″ co-doping system, the doping effect mostly depends on the single M″ doping. In the Cr-doped BaTiO_3_, an IEL emerges in the bandgap, which is quite different from the V and Nb doping systems.

In addition, we compared the band structure and DOS of V, Cr, and V-Cr doped BaTiO_3_. In the V-doped BaTiO_3_, the IELs are located at the bottom of the CB. In the Cr-doped BaTiO_3_, the IELs appear in the middle of the bandgap and form the intermediate energy level. But in the V-Cr co-doped BaTiO_3_ as shown in Figure 4, the IELs divided into two parts. One was situated at the bottom of the CB, and the other at the top of the VB. Finally, as the intermediate energy level raised from the single Cr, doping disappeared, as confirmed in Figure 5a. Thus, the V-Cr co-doping can overcome the shortcoming of the single Cr doping and combine the advantages of the V and Cr doping in reducing the bandgap to effectively shift the CBM and VBM toward the bandgap. Through such synergistic effect, the light absorption range could be expanded and the visible-light photoelectrical activity is expected to improve.

**Figure 11:**
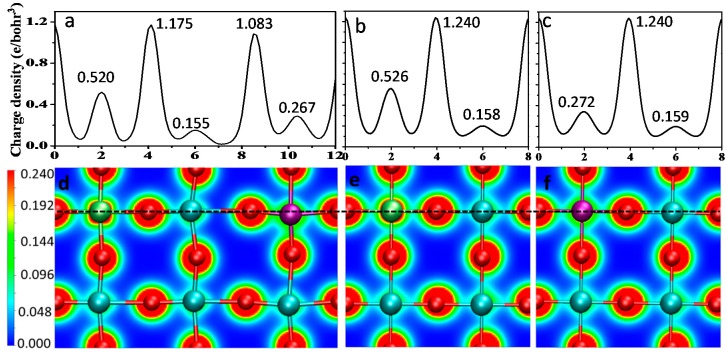
Charge-density distributions of metal doped BaTiO_3_ in (100) surfaces: (**a**–**c**) Line profiles corresponding to the black dotted lines; (**d**) V and Cr co-doped BaTiO_3_; (**e**) Cr doped BaTiO_3_; (**f**) V doped BaTiO_3_.

Furthermore, the charge density distribution was investigated for the V-doped, Cr-doped, and V-Cr co-doped BaTiO_3_ as shown in Figure 11, which indicates that Ti-O, Cr-O, and V-O form covalent bonds. Due to different electronegativity (Ti < V < Cr < O), the covalent bond strength is different. The greater the metal electronegativity is, the smaller the electronegativity difference between the metal and oxygen atoms, and the more uniform is the charge density distribution. According to an effective empirical determining method, the electronic energy level of an element is usually inversely proportional to its electronegativity. That is to say, the higher the electronegativity, the lower the position of the energy level and the lower the position of the corresponding energy band [48]. Therefore, when Cr or V with larger electronegativity was doped into BaTiO_3_, the IELs with a relatively low energy position were introduced around the CBM or in the bandgap, leading to a narrower bandgap after doping. The results are verified by the line profile of the charge density as displayed in Figure 11a–c. The charge density of Ti, O, Cr, and V is slightly decreased in various degrees after Cr-V co-doping compared to the single Cr or V doping. It can be found that in comparison to the single doping, where the charge density is more localized around the atoms, V-Cr co-doping could result in a relatively evener charge distribution. This not only causes the hybridization of the newly introduced IELs and the initial CBM or VBM of the pure BaTiO_3_, but is associated with the interaction among the newly introduced energy states due to the V-Cr synergistic effect, which accounts for the bandgap engineering by the co-doping for visible-light activation.

**Figure 12:**
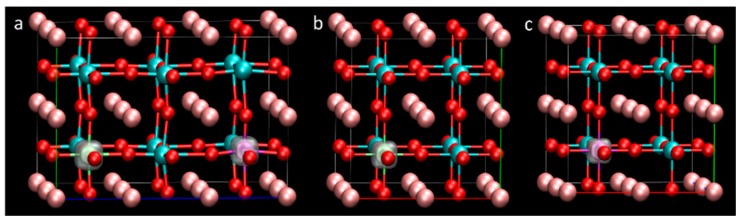
Partial charge-density distribution calculated from the top of the valence band to the bottom of the conduction band: (**a**) V and Cr co-doped BaTiO_3_; (**b**) Cr-doped BaTiO_3_; (**c**) V-doped BaTiO_3_.

In order to further analyze the charge density near the bandgap, we calculated the distribution of the partial charge density from the top of the valence band to the bottom of the conduction band, as shown in Figure 12, which could reveal the visual information of the electron densities mainly derived from the doped/co-doped atoms. The calculated partial electron density in the V-Cr co-doped BaTiO_3_ distributed around the V and Cr dopants is shown in Figure 12a, and is mainly derived from the Cr 3d isolated non-bond states and the hybrids between the V 3d and O 2p electronic states, as suggested by Figure 4 and Figure 5. Figure 12b presents the partial electron density on Cr in the Cr doped BaTiO_3_, which consists significantly of the isolated Cr 3d orbitals confirmed by Figure 10f. From Figure 12c, the partial electron density calculated in the V doped BaTiO_3_ distributed around the V atom, which primarily originates from V-O bonds arising from the hybridization of the O 2p and V 3d orbitals. As such, the doped/co-doped atoms play a key role in narrowing the bandgap of BaTiO_3_. These results also reveal that the appropriate selection of co-doping elements, such as V-Cr co-doping, can result in synergistic effects by the two co-dopants that can effectively manipulate the bandgap. Before choosing the pair of dopants for co-doping, it is important to examine the electronic structures of the individual doping and their interaction so that the pair of dopants in the co-doping system can coexist to properly modify the bandgap edge and improve the visible-light adsorption properties. Besides the electronic structure, charge mobility and surface properties/states also play important roles in the photoelectric behavior, which should be addressed in the future.

## 4. Conclusions

In summary, the formation energy and electronic properties of the transition metal V-M″ and Nb-M″ co-doped BaTiO_3_ have been studied by first-principles calculations. The results indicate that the V-M″ co-doping is more suitable than the Nb-M″ co-doping for narrowing the bandgap of BaTiO_3_ and increasing the absorption of visible light, since the V-M″ co-doping can form shallow levels around the bandgap edge while those energy levels induced by the Nb-M″ co-doping are usually deep levels in the energy band or bandgap. The optimal co-doping model is proposed as the V-Cr co-doped BaTiO_3_, which not only has favorable defect formation energy, but also significantly reduce the bandgap. Simultaneous introduction of V and Cr results in a synergistic effect, which has been demonstrated by the detailed analysis of the density of states, band structure, and charge-density distribution of the doping systems. V-Cr co-doping could overcome the shortcoming of the single Cr doping and combine the advantages of both the V and Cr doping. For this optimal co-doped BaTiO_3_ system, the Cr 3d states are located just above the VBM and the V 3d states occupied band just below the CBM, corresponding to a bandgap narrowing of about 0.4 eV, which is beneficial for efficient visible-light adsorption. The results can provide an important guideline for future experiments to modify the wide BaTiO_3_ bandgap for visible-light driven solar applications.

## Supplementary Materials

The following are available online at http://www.mdpi.com/2079-4991/8/7/455/s1, Figure S1: The lattice constants of transition metals co-doped cubic BaTiO_3_, Table S1: The special K points coordinates, Table S2: The average bond length obtained after optimization of the V-M″ co-doping system, Table S3: The average bond length obtained after optimization of the Nb-M″ co-doping system.
