# Supplementary material for: Tailoring Bandgap of Perovskite BaTiO3 by Transition Metals Co-Doping for Visible-Light Photoelectrical Applications: A First-Principles Study
Source: Nanomaterials (Basel). 2018 Jun 21;8(7):455. doi: 10.3390/nano8070455 (PMC6071297; doi:10.3390/nano8070455)
Supplement: Supplementary file 1 [file nanomaterials-08-00455-s001.pdf]

Supporting Information

Tailoring band gap of perovskite $BaTiO_3$ by transition metals co-doping for visible-light photoelectrical applications: A first principle study

Fan Yang[1,2], Liang Yang[2], Changzhi Ai[1,2], Pengcheng Xie[1,2], Shiwei Lin[1,2*], Cai-Zhuang Wang[3], Xihong Lu[4]

[1] State Key Laboratory of Marine Resource Utilization in South China Sea, Hainan University, Haikou 570228, P. R. China; 18789276621@163.com(F.Y.); changzhiai@outlook.com(C.A.); jxxpc199406@163.com (P.X.)

[2] College of Materials and Chemical Engineering, Hainan University, Haikou 570228, P. R. China; yl5923@sina.com.cn (L.T)

[3] Ames Laboratory-U. S. Department of Energy, and Department of Physics and Astronomy, Iowa State University, Ames, IA 50011, USA; wangcz@ameslab.gov

[4] School of Chemistry, Sun Yat-Sen University, Guangzhou 510275, P. R. China;Luxh6@mail.sysu.edu.cn

* Correspondence: linsw@hainu.edu.cn; Tel.: +86 898 66276115

Table S1. The special K points coordinates.

X→R→M→ Γ →R				Γ→F→Q→Z→Γ			
X	0.5	0.0	0.0	Γ	0.0	0.0	0.0
R	0.5	0.5	0.5	F	0.0	0.5	0.0
M	0.5	0.5	0.0	Q	0.0	0.5	0.5
Γ	0.0	0.0	0.0	Z	0.0	0.0	0.5
R	0.5	0.5	0.5	Γ	0.0	0.0	0.0

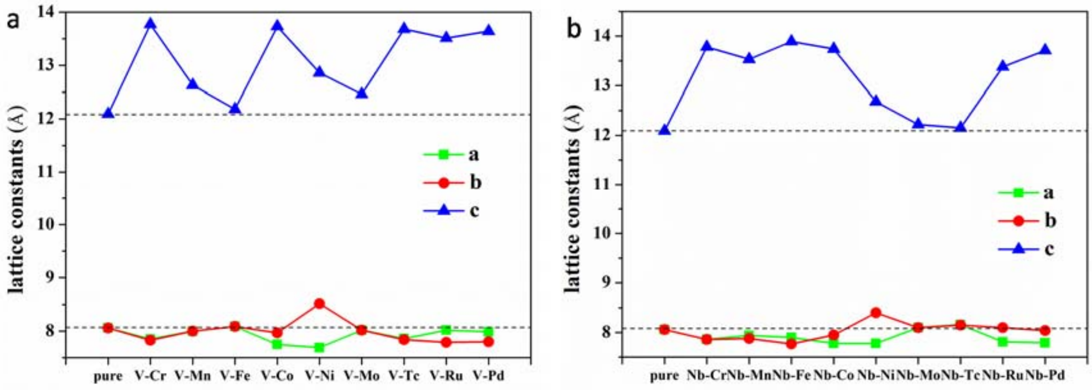

Figure S1. The lattice constants of transition metals co-doped cubic $BaTiO_3$. (a) V-M″ (Cr, Mn, Fe, Co, Ni, Mo, Tc, Ru and Pd) co-doping systems; (b) Nb-M″ (Cr, Mn, Fe, Co, Ni, Mo, Tc, Ru and Pd) co-doping systems.

Table S2. The average bond length obtained after optimization of the V-M″ co-doping system.

**Co-doping system/pure $BaTiO_3$**	**Average bond length （Ti-O）/ Å**	**Average bond length （V-O）/ Å**	**Average bond length （M″-O）/ Å**
Pure $BaTiO_3$	2.0179	-	-
V-Cr	2.0562	1.941	2.0557
V-Mn	2.0611	1.9228	2.1472
V-Fe	2.0497	1.9392	2.1245
V-Co	2.0414	1.9457	2.0355
V-Ni	2.0576	1.9472	2.1334
V-Mo	2.0511	2.0410	1.9954
V-Tc	2.0307	1.9614	2.0583
V-Ru	2.0351	1.9458	2.0855
V-Pd	2.0436	1.9299	2.1814

Table S3. The average bond length obtained after optimization of the Nb-M″ co-doping system.

Co-doping system/pure $BaTiO_3$	Average bond length （Ti-O）/ Å	Average bond length （Nb-O）/ Å	Average bond length （M″-O）/ Å
Pure $BaTiO_3$	2.0179	-	-
Nb-Cr	2.0582	2.0143	2.0628
Nb-Mn	2.0632	2.0024	2.1507
Nb-Fe	2.0482	2.0046	2.1095
Nb-Co	2.0370	2.0193	2.0291
Nb-Ni	2.0579	2.0197	2.1074
Nb-Mo	2.0313	2.0135	2.0320
Nb-Tc	2.0325	2.0152	2.0679
Nb-Ru	2.0341	2.0140	2.0804
Nb-Pd	2.0466	2.0095	2.1820
